# Synthesis, Spectroscopic Identification and Molecular Docking of Certain *N*-(2-{[2-(1*H*-Indol-2-ylcarbonyl) hydrazinyl](oxo)acetyl}phenyl)acetamides and *N*-[2-(2-{[2-(Acetylamino)phenyl](oxo)acetyl}hydrazinyl)-2-oxoethyl]-1*H*-indole-2-carboxamides: New Antimicrobial Agents

**DOI:** 10.3390/molecules23051043

**Published:** 2018-04-29

**Authors:** Maha S. Almutairi, Azza S. Zakaria, Reem I. Al-Wabli, I. Hubert Joe, Ali S. Abdelhameed, Mohamed I. Attia

**Affiliations:** 1 Department of Pharmaceutical Chemistry, College of Pharmacy, King Saud University, P.O. Box 2457, Riyadh 11451, Saudi Arabia; malmutbiri@ksu.edu.sa (M.S.A.); ralwabli@ksu.edu.sa (R.I.A.-W.); asaber@ksu.edu.sa (A.S.A.); 2 Department of Microbiology and Immunology, Faculty of Pharmacy, Alexandria University, Alexandria 21500, Egypt; azzazakaria@yahoo.com; 3 Centre for Molecular and Biophysics Research, Department of Physics, Mar Ivanios College, Thiruvananthapuram 695 015, Kerala, India; hubertjoe@gmail.com; 4 Medicinal and Pharmaceutical Chemistry Department, Pharmaceutical and Drug Industries Research Division, National Research Centre (ID: 60014618), El Bohooth Street, Dokki, Giza 12622, Egypt

**Keywords:** indole, *N*-Acetylisatins, ring opening, antimicrobial, glyoxylamides

## Abstract

*N*-(2-{[2-(1*H*-Indol-2-ylcarbonyl)hydrazinyl](oxo)acetyl}phenyl)acetamides (**5a–h**) and *N*-[2-(2-{[2-(acetylamino)phenyl](oxo)acetyl}hydrazinyl)-2-oxoethyl]-1*H*-indole-2-carboxamides (**5i–l**) were synthesized and characterized with different analytical tools. *N*-Acetylisatines **4a–d** were subjected to ring opening at their C2 carbons with the aid of different indole-bearing hydrazides **3a,b** and **7** to afford the respective glyoxylamides **5a–l**. The antimicrobial activity of the target compounds **5a–l** was assessed with the aid of Diameter of the Inhibition Zone (DIZ) and Minimum Inhibitory Concentration (MIC) assays against a panel of Gram-positive and Gram-negative bacteria and certain fungal strains. The antimicrobial screening revealed that *Staphylococcus aureus*, *Escherichia coli*, *Pseudomonas aeruginosa,* and *Candida albicans* are the most sensitive microorganisms towards the synthesized compounds **5a–l**. In addition, compounds **5c** and **5h** emerged as the most active congeners towards *Staphylococcus aureus* and *Candida albicans*, respectively. Molecular docking studies revealed the possible binding mode of compounds **5c** and **5h** to their target proteins.

## 1. Introduction

Indole is a hetero-aromatic bicyclic ring system and indoles represent an important class in drug discovery and development process [1]. Indole-bearing compounds are commonly identified and isolated from natural resources and are widely used as precursors in fine organic synthesis to develop new pharmacological lead pharmaceuticals across a broad range of therapeutic areas [2,3,4]. Biological activities exhibited by different indole derivatives include anti-inflammatory [5], anticancer [6], antihypertensive [7], antiviral [8], antibacterial [9] and antifungal activities [10].

In the same vein, 5-methoxyindole constitutes the backbone of the natural hormone melatonin, which helps sleep regulation and wake cycles through manipulation of three different melatoninergic receptors (M_1_–M_3_) [11,12]. Melatonin exhibits various therapeutic applications like anti-inflammatory, antioxidant [13], and antitumor activities [14]. In addition, 5-methoxyindole fragment was incorporated in a number of bioactive melatoninergic ligands [15,16].

2,3-Dioxindole (isatin) is another heterocyclic aromatic nucleus that was identified as an endogenous compound in humans and other mammals [17]. Isatin has a broad synthetic utility owing to its incorporation of reactive function groups which have been functionalized to prepare diverse bioactive molecules such as anticancers [18], anticonvulsants [19], and antimicrobials [20]. Moreover, *N*-acetylisatins are privileged structures in drug discovery and development processes due to their facile utility to prepare the corresponding glyoxylamides *via* attacking their C2-carbonyl functionality with different nucleophilic amines. The presence of two carbonyl groups with two different spatial orientations in glyoxylamides significantly enhances their H-bonding with protein targets and hence improving their biological activities. In addition, glyoxylamide derivatives have broad applications in organic chemistry and they are incorporated in a vast of bioactive molecules [21,22,23,24].

Therefore, the aforementioned premises encouraged us to synthesize the title glyoxylamides **5a–l** *via* opening certain *N*-acetylisatines **4a–d** with different indole-bearing hydrazides **3a,b** and **7**. The antimicrobial profile of the title compounds **5a–l** was in vitro evaluated against a panel of microorganisms including Gram-positive and Gram-negative bacteria as well as filamentous and non-filamentous fungi.

## 2. Results and Discussion

### 2.1. Chemistry

**Scheme 1:**
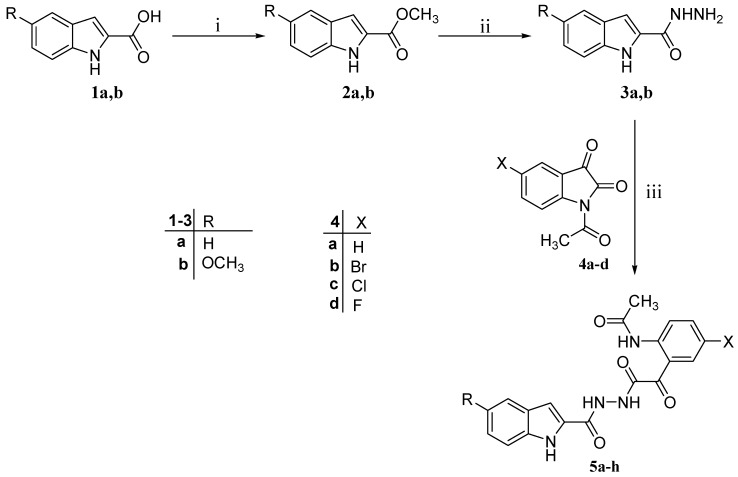
Synthesis of the target compounds **5a–h**. Reagents and conditions: (i) Methanol, drops of H_2_SO_4_, reflux, 4 h; (ii) Methanol, H_2_N-NH_2_.H_2_O, reflux, 2 h; and (iii) Acetonitrile, reflux 2 h.

**Scheme 2:**
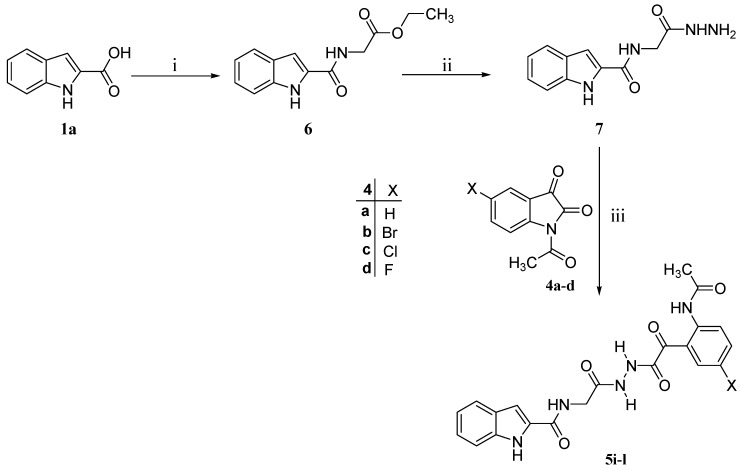
Synthesis of the target compounds **5i–l**. Reagents and conditions: (i) Tetrahydrofuran, ethyl glycinate hydrochloride, carbonyldiimidazole, rt, 18 h; (ii) Methanol, H_2_N-NH_2_.H_2_O, reflux, 2 h; and (iii) Acetonitrile, reflux 2 h.

The target compounds **5a–l** were successfully achieved as portrayed in Scheme 1 and Scheme 2. Thus, *N*-acetylisatines **4a–d** were allowed to react with the appropriate hydrazide **3a,b** in acetonitrile, a polar non-nucleophile solvent, to afford the respective targets **5a–h** (Scheme 1). NMR (^1^H and ^13^C) as well as mass spectral data of compounds **5a–h** are consistent with their proposed chemical structures. The single crystal X-ray structure of compound **5c** [25], as a representative example of compounds **5a–h**, confirmed doubtlessly the assigned chemical structures of **5a–h**.

Ethyl glycinate was coupled with the commercially available indole-2-carboxylic acid (**1a**) in the presence of carbonyldiimidazole to furnish the coupled product **6** (Scheme 2). Hydrazinolysis of the ester functionality of compound **6** with hydrazine hydrate yielded the respective hydrazide **7**. Subsequently, *N*-acetylisatines **4a–d** were allowed to react with hydrazide **7** to give the corresponding target compounds **5i–l** (Scheme 2). The assigned chemical structures of compounds **5i-l** were confirmed via their NMR (^1^H and ^13^C) and mass spectral data.

### 2.2. Antimicrobial Evaluation

The title glyoxylamides **5a–l** were divided into two sets, the first set contains compounds **5a–h** in which either indole hydrazide **3a** or 5-methoxyindole hydrazide **3b** was used for ring opening of *N*-acetylisatin derivatives **4a–d**. The second set comprises compounds **5i–l** in which the hydrazide **7** was used for ring opening of compounds **4a–d**.

Table 1 presented the results of the preliminary antimicrobial activity of the title compounds **5a–l** against certain Gram-positive and Gram-negative bacteria as well as certain fungi using Diameter of the Inhibition Zone (DIZ) assay. Compounds **5c** and **5h** manifested the best activity against the tested Gram-positive bacteria with DIZ values of 21 and 22 mm towards *S. aureus* and *B. subtilis*, respectively. On the other hand, compounds **5b** and **5f** showed the best activity against the tested Gram-negative bacteria with DIZ values of 19 and 18 mm against *E. coli* and *Ps. Aeruginosa*, respectively. *C. albicans* was the most sensitive fungus towards compound **5h** with DIZ value of 25 mm.

The results of the Minimum Inhibitory Concentration (MIC) assay for the target compounds **5a–l** are presented in Table 2. *S. aureus* is the most sensitive Gram-positive bacteria towards the tested compounds **5a–l** with MIC values of 3.9, 31.25, and 62.5 μg/mL for compounds **5c**, **5d** and **5b** or **5i**, respectively. Also, *B. subtilis* was sensitive to compound **5h** with MIC value of 62.5 μg/mL. Regarding the tested strains of Gram-negative bacteria, compounds **5d**, **5i,** and **5k** are the best candidates towards *E. coli* (compounds **5i** and **5k**) and compound **5d** towards *Ps. Aeruginosa* being equipotent with an MIC value of 62.5 μg/mL. Compound **5h** manifested the best antifungal profile for the whole synthesized series **5a–l** as it showed MIC values of 7.8, 31.25, and 62.5 μg/mL against *C. albicans*, *A. niger,* and *P. notatum*, respectively. 

In summary, it can be deduced from the above antimicrobial screening tests that *S. aureus*, *E. coli*, *Ps. Aeruginosa,* and *C. albicans* are the most sensitive microorganisms towards the synthesized compounds **5a–l**. Compound **5c** bearing indole hydrazide **3a** fragment and a chloro substituent in the first set **5a–h**, is the best candidate against *S. aureus*. In the same set, compound **5h** bearing 5-methoxyindole hydrazide **3b** fragment and a fluoro substituent and is the most active congener towards both *B. subtilis* and the tested three fungal strains. On the other hand, compounds **5i** and **5k,** bearing indole hydrazide **7** fragment in the second set **5i–l**, are the most active compounds against *E. coli* being equipotent, while compound **5d** is the most active candidate towards *Ps. Aeruginosa.*

### 2.3. Molecular Docking

**Figure 1:**
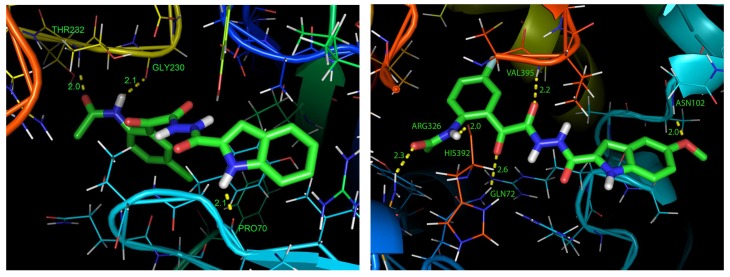
Hydrogen bonding projection of protein-ligand interactions and binding pose for compound **5c** (left) and compound **5h** (right).

The three-dimensional (3D) structural coordinates of compounds **5c** and **5h** were sketched by ChemDraw Ultra 7.0.1 program [26]. The energy of compounds **5c** and **5h** was minimized using PRODRG online server on basis of GROMACS force field method [27]. The antibacterial (PDB ID: 4DH6) [28] and antifungal (PDB ID: 1EA1) [29] target proteins have been chosen for the docking study for compounds **5c** and **5h**, respectively. The 3D structural coordinates of the target proteins were downloaded from the RCSB protein data bank [30]. The target proteins manipulation has been carried out by following steps: (i) all water molecules were removed; (ii) hydrogen atoms were added to the crystal structure; (iii) Kollaman′s charges were added; and (iv) the docked inhibitors were removed from the target proteins. The protein (rigid) and ligand (flexible) docking was performed with the aid of AutoDock 4.2 [31] program interfaced with MGL Tools 1.5.6 rc3 [32] to create affinity grids centered on the active site with 90 × 90 × 90 grid size. The results of the predicted free binding energy (∆*E*), inhibition constant (*K*_i_) as well as the bounded amino acid resides of the complex are given in Table 3. The docking results were evaluated by sorting the free binding energies predicted by their docking conformations. The best conformation binding energy is predicted to be –7.88 kcal/mol with *K*_i_ value of 1.67 μM for compound **5c**. Its hydrogen bonding interactions were observed with THR232, GLY230, and PRO70 amino acid residues of 4DH6 target protein (Figure 1).

On the other hand, the best conformation binding energy is predicted to be –7.28 kcal/mol with *K*_i_ value of 4.63 μM for compound **5h**. Its hydrogen bonding interactions were noted with ARG326, HIS392, GLN72, VAL395, and ASN102 amino acid residues of 1EA1 target protein (Figure 1). The molecular docking investigations manifested the possible binding pose of compounds **5c** and **5h** inside their target bacterial and fungal proteins, respectively.

## 3. Experimental

### 3.1. General

A Gallenkamp device was used to measure melting points and they are uncorrected. Bruker NMR spectrometer (Bruker, Reinstetten, Germany) was used to record the NMR spectra of the synthesized compounds **5a–l** in DMSO-*d*_6_ at 500 MHz for ^1^H and 125.76 MHz for ^13^C at the Research Center, College of Pharmacy, King Saud University, Saudi Arabia. Chemical shifts are expressed in δ-values (ppm) relative to TMS as an internal standard. Elemental analyses were carried out at Microanalysis Laboratory, Cairo University, Cairo, Egypt and the results agreed favorably with the proposed structures within ± 0.4% of the theoretical values. Agilent Quadrupole 6120 LC/MS with ESI (Electrospray ionization) source (Agilent Technologies, Palo Alto, CA, USA) was used to record mass spectra of the synthesized compounds. High-resolution mass spectrometry (HR-MS) measurements were performed on an LTQ-Orbitrap XL coupled to matrix-assisted laser desorption ionization (MALDI). Compounds **2a,b** [33], **3a,b** [34], **4a–d** [21], and **6** [35] were prepared according to literature procedures. Ampicillin (AMP) was obtained from Sigma-Aldrich Co. (St. Louis, MO, USA) and fluconazole (FLC) was purchased from Shouguang-Fukang Pharmaceutical Ltd. (Shandong, China).

### 3.2. Chemistry

Methyl 1*H*-indole-2-carboxylate (**2a**): White powder; melting point (m.p.) 150–151 °C [33].

Methyl 5-methoxy-1*H*-indole-2-carboxylate (**2b**): Yellow powder; m.p. 176–177 °C [36].

1*H*-Indole-2-carbohydrazide (**3a**): Off-White powder; m.p. 251–253 °C [37].

5-Methoxy-1*H*-indole-2-carbohydrazide (**3b**): Off-White powder; m.p. 266–268 °C [38].

1-Acetyl-1*H*-indole-2,3-dione (**4a**): Yellow crystals; m.p. 141–143 °C [39].

1-Acetyl-5-bromo-1*H*-indole-2,3-dione (**4b**): Brown powder; m.p. 167–169 °C [21].

1-Acetyl-5-chloro-1*H*-indole-2,3-dione (**4c**): Light brown powder; m.p. 240–242 °C [40].

1-Acetyl-5-fluoro-1*H*-indole-2,3-dione (**4d**): Yellow powder; m.p. 147-149 °C [21].

#### 3.2.1. General Procedure for the Synthesis of the Target Compounds **5a–h**

The appropriate *N*-acetylisatin **4a–d** (1 mmol) was added to a suspension containing the proper acid hydrazide **3a,b** (1 mmol) in acetonitrile (15 mL). The reaction mixture was heated to reflux for two hours, cooled to room temperature, and filtered. The collected solid was dried and re-crystallized from ethanol to give the title compounds **5a–h**. 

*N*-(2-{[2-(1*H*-Indol-2-ylcarbonyl)hydrazinyl](oxo)acetyl}phenyl)acetamide (**5a**): Yellow powder; m.p. 249–250 °C (yield 58%); ^1^H-NMR (DMSO-*d*_6_): δ (ppm) 2.20 (s, 3H, C*H*_3_), 7.09 (t, *J* = 7.5 Hz, 1H, Ar-H), 7.24 (t, *J* = 7.5 Hz, 1H, Ar-H), 7.27 (d, *J* = 1.5 Hz, 1H, CH-3-indole), 7.31 (t, *J* = 7.5 Hz, 1H, Ar-H), 7.47 (d, *J* = 8.5 Hz, 1H, Ar-H), 7.68 (d, *J* = 8.0 Hz, 1H, Ar-H), 7.73 (dd, *J* = 1.5, 8.5 Hz, 1H, Ar-H), 8.11 (dd, *J* = 1.0, 8.0 Hz, 1H, Ar-H), 8.21 (d, *J* = 8.0 Hz, 1H, Ar-H), 10.71 (s, 1H, NH), 10.75 (s, 1H, NH), 10.90 (s, 1H, NH), 11.85 (s, 1H, NH-indole); ^13^C-NMR (DMSO-*d*_6_): δ (ppm) 24.9 (*C*H_3_), 104.2, 112.9, 120.5, 121.5, 121.9, 122.3, 123.6, 124.4, 127.4, 129.5, 133.5, 135.9, 137.3, 140.3 (Ar-CH and Ar-C), 158.3, 164.5, 169.6, 192.7 (4× C=O); MS *m*/*z* (ESI): 363 [M − H]^−^; HR-MS (MALDI) calcd for C_19_H_16_N_4_O_4_: 363.1093, found: 363.1028 (M − H). 

*N*-(4-Bromo-2-{[2-(1*H*-indol-2-ylcarbonyl)hydrazinyl](oxo)acetyl}phenyl)acetamide (**5b**): Yellow powder; m.p. 261–263 °C (yield 56%); ^1^H-NMR (DMSO-*d*_6_): δ (ppm) 2.19 (s, 3H, C*H*_3_), 7.08 (t, *J* = 7.5 Hz, 1H, Ar-H), 7.24 (t, *J* = 7.5 Hz, 1H, Ar-H), 7.27 (d, *J* = 1.5 Hz, 1H, CH-3-indole), 7.49 (d, *J* = 8.5 Hz, 1H, Ar-H), 7.68 (d, *J* = 8.0 Hz, 1H, Ar-H), 7.89 (dd, *J* = 2.0, 8.5 Hz, 1H, Ar-H), 7.96 (d, *J* = 8.5 Hz, 1H, Ar-H), 8.08 (d, *J* = 2.5 Hz, 1H, Ar-H), 10.65 (s, 1H, NH), 10.68 (s, 1H, NH), 10.91 (s, 1H, NH), 11.83 (s, 1H, NH-indole); ^13^C-NMR (DMSO-*d*_6_): δ (ppm) 24.7 (*C*H_3_), 104.3, 112.9, 114.0, 115.4, 120.2, 122.3, 123.7, 124.4, 127.3, 129.5, 133.4, 134.6, 137.5, 138.6 (Ar-CH and Ar-C), 158.3, 160.6, 169.4, 192.6 (4× C=O); MS *m*/*z* (ESI): 441 [M − H]^−^, 442 [(M + 1) − H]^−^, 443[(M + 2) − H]^−^; HR-MS (MALDI) calcd for C_19_H_15_BrN_4_O_4_: 441.0198, found: 441.0206 (M − H).

*N*-(4-Chloro-2-{[2-(1*H*-indol-2-ylcarbonyl)hydrazinyl](oxo)acetyl}phenyl)acetamide (**5c**): Yellow powder; m.p. 259–260 °C (yield 77%); ^1^H-NMR (DMSO-*d*_6_): δ (ppm) 2.19 (s, 3H, C*H*_3_), 7.08 (t, *J* = 7.5 Hz, 1H, Ar-H), 7.24 (t, *J* = 7.5 Hz, 1H, Ar-H), 7.27 (d, *J* = 1.5 Hz, 1H, CH-3-indole), 7.48 (d, *J* = 8.0 Hz, 1H, Ar-H), 7.68 (d, *J* = 8.0 Hz, 1H, Ar-H), 7.77 (dd, *J* = 2.5, 8.5 Hz, 1H, Ar-H), 7.99 (d, *J* = 2.5 Hz, 1H, Ar-H), 7.02 (d, *J* = 8.5 Hz, 1H, Ar-H), 10.66 (s, 1H, NH), 10.69 (s, 1H, NH), 10.91 (s, 1H, NH), 11.83 (s, 1H, NH-indole); ^13^C-NMR (DMSO-*d*_6_): δ (ppm) 24.7 (*C*H_3_), 104.3, 112.9, 120.5, 122.3, 123.8, 124.4, 124.8, 127.4, 127.7, 129.5, 131.7, 134.9, 137.2, 138.2 (Ar-CH and Ar-C), 160.9, 163.4, 169.7, 190.2 (4 × C=O); MS *m*/*z* (ESI): 397 [M − H]^−^, 398 [(M + 1) − H]^−^, 399 [(M + 2) − H]^−^; HR-MS (MALDI) calcd for C_19_H_15_ClN_4_O_4_: 397.0704, found: 397.0738 (M − H). 

*N*-(4-Fluoro-2-{[2-(1*H*-indol-2-ylcarbonyl)hydrazinyl](oxo)acetyl}phenyl)acetamide (**5d**): Yellow powder; m.p. 268–270 °C(yield 73%); ^1^H-NMR (DMSO-*d*_6_): δ (ppm) 2.17 (s, 3H, C*H*_3_), 7.08 (t, *J* = 7.5 Hz, 1H, Ar-H), 7.24 (t, *J* = 8.0 Hz, 1H, Ar-H), 7.27 (d, *J* = 1.5 Hz, 1H, CH-3-indole), 7.47 (d, *J* = 8.5 Hz, 1H, Ar-H), 7.60 (ddd, *J* = 2.5, 3.0, 8.5 Hz, 1H, Ar-H), 7.68 (d, *J* = 8.0 Hz, 1H, Ar-H), 7.84 (dd, *J* = 3.0, 9.5 Hz, 1H, Ar-H), 7.97–7.99 (m, 1H, Ar-H), 10.55 (s, 1H, NH), 10.69 (s, 1H, NH), 10.89 (s, 1H, NH), 11.85 (s, 1H, NH-indole); ^13^C-NMR (DMSO-*d*_6_): δ (ppm) 24.5 (*C*H_3_), 104.3, 112.9, 118.4, 118.5, 120.5, 122.2, 122.3, 124.4, 127.4, 129.5, 130.4, 133.2, 135.6, 137.7 (Ar-CH and Ar-C), 161.1, 163.6, 169.6, 190.1 (4× C=O); MS *m*/*z* (ESI): 381 [M − H]^−^; HR-MS (MALDI) calcd for C_19_H_15_FN_4_O_4_: 381.0999, found: 381.0951 (M − H).

*N*-{2-[{2-[(5-Methoxy-1*H*-indol-2yl)carbonyl]hydrazinyl}(oxo)acetyl]phenyl}acetamide (**5e**): Pale yellow powder; m.p. 228-230 °C (yield 52%); ^1^H-NMR (DMSO-*d*_6_): δ (ppm) 2.19 (s, 3H, C*H*_3_), 3.78 (s, 3H, OC*H*_3_), 6.90 (dd, *J* = 2.5, 8.5 Hz, 1H, Ar-H), 7.15 (d, *J* = 1.0 Hz, 1H, Ar-H), 7.19 (s, 1H, CH-3-indole), 7.31(t, *J* = 7.5 Hz, 1H, Ar-H), 7.35 (d, *J* = 9.0 Hz, 1H, Ar-H), 7.72 (t, *J* = 8.0 Hz, 1H, Ar-H), 8.11 (d, *J* = 8.0 Hz, 1H, Ar-H), 8.20 (d, *J* = 8.5 Hz, 1H, Ar-H), 10.66 (s, 1H, NH), 10.75 (s, 1H, NH), 10.89 (s, 1H, NH), 11.71 (s, 1H, NH-indole); ^13^C-NMR (DMSO-*d*_6_): δ (ppm) 24.9 (*C*H_3_), 55.7 (O*C*H_3_), 102.6, 103.9, 113.7, 115.8, 121.5, 121.9, 123.6, 127.8, 129.8, 132.5, 133.5, 135.9, 140.3, 154.4 (Ar-CH and Ar-C), 160.9, 164.5, 169.6, 192.7 (4× C=O); MS *m*/*z* (ESI): 393 [M − H]^−^; HR-MS (MALDI) calcd for C_20_H_18_N_4_O_5_: 393.1199, found: 393.1139 (M − H).

*N*-{4-Bromo-2-[{2-[(5-methoxy-1*H*-indol-2-yl)carbonyl]hydrazinyl}(oxo)acetyl]phenyl}acetamide (**5f**): Light brown powder; 248–250 °C (yield 65%); ^1^H-NMR (DMSO-*d*_6_): δ (ppm) 2.18 (s, 3H, C*H*_3_), 3.78 (s, 3H, OC*H*_3_), 6.89 (dd, *J* = 2.5, 9.0 Hz, 1H, Ar-H), 7.14 (d, *J* = 2.0 Hz, 1H, Ar-H), 7.19 (d, *J* = 1.5 Hz, 1H, CH-3-indole), 7.36 (d, *J* = 8.5 Hz, 1H, Ar-H), 7.89 (dd, *J* = 2.0, 8.5 Hz, 1H, Ar-H), 7.97 (d, *J* = 9.0 Hz, 1H, Ar-H), 8.08 (d, *J* = 2.5 Hz, 1H, Ar-H), 10.62 (s, 1H, NH), 10.65 (s, 1H, NH), 10.88 (s, 1H, NH), 11.68 (s, 1H, NH-indole); ^13^C-NMR (DMSO-*d*_6_): δ (ppm) 24.7 (*C*H_3_), 55.7 (O*C*H_3_), 102.5, 103.9, 113.7, 115.4, 115.7, 123.9, 125.0, 127.7, 129.8, 132.5, 134.5, 137.8, 138.6, 154.4 (Ar-CH and Ar-C), 160.9, 163.4, 169.7, 190.1 (4× C=O); MS *m*/*z* (ESI): 471 [M − H]^−^, 472 [(M + 1) − H]^−^, 473 [(M + 2) − H]^−^; HR-MS (MALDI) calcd for C_20_H_17_BrN_4_O_5_: 471.0304, found: 471.0365 (M − H).

*N*-{4-Chloro-2-[{2-[(5-methoxy-1*H*-indol-2-yl)carbonyl]hydrazinyl}(oxo)acetyl]phenyl}acetamide (**5g**): Yellow powder; 255–257 °C (yield 77%); ^1^H-NMR (DMSO-*d*_6_): δ (ppm) 2.18 (s, 3H, C*H*_3_), 3.78 (s, 3H, OC*H*_3_), 6.89 (dd, *J* = 2.0, 9.0 Hz, 1H, Ar-H), 7.14 (d, *J* = 2.5 Hz, 1H, Ar-H), 7.19 (d, *J* = 2.0 Hz, 1H, CH-3-indole), 7.36 (d, *J* = 8.5 Hz, 1H, Ar-H), 7.78 (dd, *J* = 2.5, 9.0 Hz, 1H, Ar-H), 7.99 (d, *J* = 2.5 Hz, 1H, Ar-H), 8.03 (d, *J* = 9.0 Hz, 1H, Ar-H), 10.63 (s, 1H, NH), 10.65 (s, 1H, NH), 10.89 (s, 1H, NH), 11.68 (s, 1H, NH-indole); ^13^C-NMR (DMSO-*d*_6_): δ (ppm) 24.7 (*C*H_3_), 55.7 (O*C*H_3_), 102.5, 103.9, 113.7, 115.7, 123.8, 124.7, 127.6, 127.7, 129.7, 131.7, 132.6, 134.9, 138.3, 154.4 (Ar-CH and Ar-C), 160.9, 163.5, 169.7, 190.2 (4× C=O); MS *m*/*z* (ESI): 427 [M − H]^−^, 428 [(M + 1) − H]^−^, 429 [(M + 2) − H]^−^; HR-MS (MALDI) calcd for C_20_H_17_ClN_4_O_5_: 427.0809, found: 427.0845 (M − H).

*N*-{4-Fluoro-2-[{2-[(5-methoxy-1*H*-indol-2-yl)carbonyl]hydrazinyl}(oxo)acetyl]phenyl}acetamide (**5h**): Yellow powder; 238–240 °C (yield 60%); ^1^H-NMR (DMSO-*d*_6_): δ (ppm) 2.17 (s, 3H, C*H*_3_), 3.78 (s, 3H, OC*H*_3_), 6.89 (dd, *J* = 2.5, 9.0 Hz, 1H, Ar-H), 7.14 (d, *J* = 2.0 Hz, 1H, Ar-H), 7.18 (d, *J* = 1.5 Hz, 1H, CH-3-indole), 7.35 (d, *J* = 9.0 Hz, 1H, Ar-H), 7.60 (ddd, *J* = 2.5, 3.0, 9.0 Hz, 1H, Ar-H), 7.85 (dd, *J* = 3.0, 9.0 Hz, 1H, Ar-H), 7.99 (dd, *J* = 1.0, 9.0 Hz, 1H, Ar-H), 10.55 (s, 1H, NH), 10.64 (s, 1H, NH), 10.87 (s, 1H, NH), 11.70 (s, 1H, NH-indole); ^13^C-NMR (DMSO-*d*_6_): δ (ppm) 24.5 (*C*H_3_), 55.8 (O*C*H_3_), 102.6, 103.9, 113.7, 115.8, 122.2, 122.4, 124.4, 124.5, 127.8, 129.7, 132.6, 135.9, 142.4, 154.4 (Ar-CH and Ar-C), 160.9, 163.7, 169.6, 190.3 (4× C=O); MS *m*/*z* (ESI): 411 [M − H]^−^; HR-MS (MALDI) calcd for C_20_H_17_FN_4_O_5_: 411.1105, found: 411.1148 (M − H).

Ethyl [(1*H*-indol-2-ylcarbonyl)amino]acetate (**6**): White powder; m.p. 222-224 °C [35].

#### 3.2.2. Synthesis of *N*-(2-Hydrazinyl-2-oxoethyl)-1*H*-indole-2-carboxamide (**7**)

Hydrazine hydrate (50 mmol) was added to a suspension containing compound **6** (5 mmol) in methanol (15 mL). The reaction mixture was heated to reflux for three hours under stirring. The cooled reaction mixture was filtered off and dried to furnish compound **7** in 79% yield as a white powder, m.p. 227–229 °C, which was pure enough to be used for further reactions. ^1^H-NMR (DMSO-*d*_6_) δ (ppm): 3.89 (d, *J* = 6.0 Hz, 2H, C*H*_2_), 4.52 (s, 2H, NH_2_), 7.02–7.06 (m, 1H, Ar-H), 7.10 (s, 1H, CH-3-indole), 7.16–7.30 (m, 1H, Ar-H), 7.43–7.46 (m, 1H, Ar-H), 7.60 (d, *J* = 7.7 Hz, 1H, Ar-H), 9.76 (t, *J* = 6.0 Hz, 1H, -CH_2_-N*H*), 9.80 (s, 1H, NH), 11.63 (s, 1H, NH); ^13^C-NMR (DMSO-*d*_6_) δ (ppm): 41.3 (*C*H_2_), 102.3, 112.8, 120.2, 121.9, 123.6, 127.5, 130.9, 136.8 (Ar-CH and Ar-C), 161.7, 168.8 (2× C=O); MS *m/z*: 231 [M − H]^−^; HR-MS (MALDI) calcd for C_11_H_12_N_4_O_2_: 231.0882, found: 231.0808 (M − H).

#### 3.2.3. General Procedure for the Synthesis of the Target Compounds **5i–l**

Compounds **5i–l** were prepared by adopting the aforementioned procedure for the synthesis for compounds **5a–h**.

*N*-[2-(2-{[2-(Acetylamino)phenyl](oxo)acetyl}hydrazinyl)-2-oxoethyl]-1*H*-indole-2-carboxamide (**5i**): Pale yellow powder; m.p. 228–230 °C (yield 61%); ^1^H-NMR (DMSO-*d*_6_): δ (ppm) 2.19 (s, 3H, C*H*_3_), 4.08 (d, *J* = 6.0 Hz, 2H, C*H*_2_), 7.07 (t, *J* = 7.0 Hz, 1H, Ar-H), 7.16–7.24 (m, 1H, Ar-H), 7.27 (s, 1H, CH-3-indole), 7.29–7.32 (m, 1H, Ar-H), 7.46 (t, *J* = 7.5 Hz, 1H, Ar-H), 7.62–7.68 (m, 1H, Ar-H), 7.69–7.74 (m, 1H, Ar-H), 8.11 (d, *J* = 8.0 Hz, 1H, Ar-H), 8.20 (d, *J* = 8.5 Hz, 1H, Ar-H), 8.88 (t, *J* = 6.0 Hz, 1H, -CH_2_-N*H*), 10.70 (s, 1H, NH), 10.74 (s, 1H, NH), 11.63 (s, 1H, NH), 11.85 (s, 1H, NH); ^13^C-NMR (DMSO-*d*_6_): δ (ppm) 24.9 (*C*H_3_), 63.4 (CH_2_), 103.6, 104.2, 112.9, 120.5, 122.3, 123.6, 124.4, 127.4, 127.6, 129.5, 131.8, 135.9, 136.9, 140.2 (Ar-CH and Ar-C), 161.0, 161.9, 168.7, 169.6, 192.7 (5× C=O); MS *m*/*z* (ESI): 420 [M − H]^−^; HR-MS (MALDI) calcd for C_21_H_19_N_5_O_5_: 420.1307, found: 420.1353 (M − H).

*N*-[2-(2-{[2-(Acetylamino)-5-bromophenyl](oxo)acetyl}hydrazinyl)-2-oxoethyl]-1*H*-indole-2-carboxamide (**5j**): Green-yellow powder; m.p. 238-240 °C (yield 47%); ^1^H-NMR (DMSO-*d*_6_): δ (ppm) 2.13 (s, 3H, C*H*_3_), 4.07 (d, *J* = 6.0 Hz, 2H, C*H*_2_), 7.04–7.09 (m, 1H, Ar-H), 7.19–7.24 (m, 1H, Ar-H), 7.27 (s, 1H, CH-3-indole), 7.45–7.49 (m, 1H, Ar-H), 7.64–7.69 (m, 1H, Ar-H), 7.84–7.86 (m, 1H, Ar-H), 7.89–7.95 (m, 1H, Ar-H),7.98 (d, *J* = 2.0 Hz, 1H, Ar-H), 8.89 (t, *J* = 6.0 Hz, 1H, -CH_2_-N*H*), 10.27 (s, 1H, NH), 10.59 (s, 1H, NH), 11.63 (s, 1H, NH), 11.82 (s, 1H, NH); ^13^C-NMR (DMSO-*d*_6_): δ (ppm) 24.7 (*C*H_3_), 63.4 (CH_2_), 103.6, 112.8, 115.4, 122.1, 122.3, 123.9, 124.0, 125.2, 127.5, 129.8, 131.8, 134.5, 137.7, 138.5 (Ar-CH and Ar-C), 161.9, 162.9, 168.7, 169.7, 189.9 (5× C=O); MS *m*/*z* (ESI): 498 [M − H]^−^, 499 [(M + 1) − H]^−^, 500 [(M + 2) − H]^–^; HR-MS (MALDI) calcd for C_21_H_18_BrN_5_O_5_: 498.0413, found: 498.0467 (M − H).

*N*-[2-(2-{[2-(Acetylamino)-5-chlorophenyl](oxo)acetyl}hydrazinyl)-2-oxoethyl]-1*H*-indole-2-carboxamide (**5k**): Yellow powder; m.p. 237–239 °C (yield 72%); ^1^H-NMR (DMSO-*d*_6_): δ (ppm) 2.14 (s, 3H, C*H*_3_), 4.07 (d, *J* = 6.0 Hz, 2H, C*H*_2_), 7.04–7.09 (m, 1H, Ar-H), 7.19–7.24 (m, 1H, Ar-H), 7.27 (s, CH-3-indole), 7.45–7.49 (m, 1H, Ar-H), 7.64–7.69 (m, 1H, Ar-H), 7.73–7.78 (m, 1H, Ar-H), 7.88 (d, *J* = 2.0 Hz, 1H, Ar-H),7.97–8.03 (m, 1H, Ar-H), 8.89(t, *J* = 6.0 Hz, 1H, -CH_2_-N*H*),10.59 (s, 1H, NH), 10.66 (s, 1H, NH), 11.63 (s, 1H, NH), 11.83 (s, 1H, NH); ^13^C-NMR (DMSO-*d*_6_): δ (ppm) 24.6 (*C*H_3_), 63.4 (CH_2_), 103.6, 104.3, 112.9, 122.1, 122.3, 123.8, 124.4, 127.4, 127.7, 129.5, 131.8, 134.8, 137.3, 138.2 (Ar-CH and Ar-C), 160.9, 161.9, 168.7, 169.7, 190.0 (5× C=O); MS *m*/*z* (ESI): 454 [M − H]^–^, 455 [(M + 1) − H]^–^, 456 [(M + 2) – H]^–^; HR-MS (MALDI) calcd for C_21_H_18_ClN_5_O_5_: 454.0918, found: 454.0973 (M − H).

*N*-[2-(2-{[2-(Acetylamino)-5-fluorophenyl](oxo)acetyl}hydrazinyl)-2-oxoethyl]-1*H*-indole-2-carboxamide (**5l**): Pale yellow powder m.p. 239–241 °C (yield 58%); ^1^H-NMR (DMSO-*d*_6_): δ (ppm) 2.12 (s, 3H, C*H*_3_), 4.07 (d, *J* = 6.0 Hz, 2H, C*H*_2_), 7.04–7.09 (m, 1H, Ar-H), 7.19–7.24 (m, 1H, Ar-H), 7.27 (s, 1H, CH-3-indole), 7.45–7.48 (m, 1H, Ar-H), 7.54–7.60 (m, 1H, Ar-H), 7.64–7.69 (m, 1H, Ar-H), 7.84 (dd, *J* = 9.0, 3.0 Hz, 1H, Ar-H), 7.97–7.99 (m, 1H, Ar-H), 8.88 (t, *J* = 6.0 Hz, 1H, -CH_2_-N*H*), 10.48 (s, 1H, NH), 10.55 (s, 1H, NH), 11.62 (s, 1H, NH), 11.85 (s, 1H, NH); ^13^C-NMR (DMSO-*d*_6_): δ (ppm) 24.4 (*C*H_3_), 63.1 (CH_2_), 100.0, 103.6, 112.9, 120.5, 122.3, 123.9, 124.4, 127.4, 127.5, 129.5, 131.8, 135.6, 136.9, 140.9 (Ar-CH and Ar-C), 161.0, 161.9, 168.7, 169.5, 192.8 (5× C=O); MS *m*/*z* (ESI): 438 [M – H]^–^; HR-MS (MALDI) calcd for C_21_H_18_FN_5_O_5_: 438.1213, found: 438.1261 (M − H).

### 3.3. Antimicrobial Activity

#### 3.3.1. Isolates

The common pathogenic microorganisms were selected: four Gram-positive isolates, namely *Bacillus subtilis* (*B. subtilis*), *Enterococcus fecalis* (*E. fecalis*), Methicillin resistant *Staphylococcus aureus* (MRSA), and *Staphylococcus aureus* (*S. aureus*); five Gram-negative organisms, namely *Escherichia coli* (*E. coli*), *Klebsiella pneumonia* (*K. pneumonia*), *Proteus vulgaris* (*P. vulgaris*), *Pseudomonas aeruginosa* (*Ps. Aeruginosa*), and *Salmonella enteridis* (*S. enteridis*); and three fungal isolates, namely *Asperagillus niger* (*A. niger*), *Candida albicans* (*C. albicans*), *and Penicillum notatum* (*P. notatum*). All isolates were obtained from King Khaled Hospital, Riyadh, Saudi Arabia.

#### 3.3.2. Disk Diffusion Assay

Disk diffusion assay for the title compounds **5a–l** was carried out at 1000 μg/mL concentration as previously reported [34] (Supplementary Materials: Antimicrobial Activity).

#### 3.3.3. Determination of Minimum Inhibitory Concentrations (MICs)

The MIC values for the title compounds **5a–l** and the reference compounds were determined by adopting the previously reported method [34].

## 4. Conclusions

Opening *N*-acetylisatins **4a–d** with the aid of different hydrazides **3a,b** and/or **7** has been successfully achieved to furnish the corresponding glyoxylamides **5a–l**. The new acetamides **5a–h** and carboxamides **5i–l** were characterized with various spectroscopic techniques. In vitro antimicrobial potential of the title glyoxylamides **5a–l** was examined using DIZ and MIC assays towards a panel of Gram-positive and Gram-negative bacteria as well as filamentous and non filamentous fungi. *S. aureus*, *E. coli*, *Ps. Aeruginosa,* and *C. albicans* are the most sensitive microorganisms towards the synthesized compounds **5a–l**. Compounds **5b–d** bearing indole hydrazide **3a** fragment are the most active candidates towards *S. aureus*. Compound **5h** bearing 5-methoxyindole hydrazide **3b** moiety manifested the best antifungal profile against the tested three fungal strains being about three-fold more potent than fluconazole. Compounds **5i** and **5k** bearing indole hydrazide **7** fragment are the most active congeners against *E. coli* being equipotent with MIC value of 62.5 μg/mL. Molecular docking investigations predicted the possible binding pose of compounds **5c** and **5h** to their target proteins. It is believed that the results of the current investigation could support the development of new indole-based bioactive glyoxylamides.

## Figures and Tables

**Table:** 

**Compound No.**	**R**	**X**
**5a**	H	H
**5b**	H	Br
**5c**	H	Cl
**5d**	H	F
**5e**	OCH_3_	H
**5f**	OCH_3_	Br
**5g**	OCH_3_	Cl
**5h**	OCH_3_	F

**Table:** 

**Compound No.**	**R**	**X**
**5i**	H	H
**5j**	H	Br
**5k**	H	Cl
**5l**	H	F

**Table 1:** Diameter of the Inhibition Zone (DIZ) of the title compounds **5a–l**, AMP, and FLC against Gram-positive bacteria, Gram-negative bacteria, and fungi.

Compound No.	DIZ in mm ± S.D.*											
	Strain											
	Gram-Positive Bacteria				Gram-Negative Bacteria					Fungi		
	*B.* *Subtilis*	*E.* *Fecalis*	MRSA	*S.* *Aureus*	*E.* *Coli*	*K. Pneumonia*	*P.* *Vulgaris*	*Ps. Aeruginosa*	*S.* *Enteridis*	*A.* *Niger*	*C. Albicans*	*P.* *Notatum*
**5a**	9 ± 0.0	9 ± 1.0	9 ± 0.0	19 ± 0.0	13 ± 0.9	−ve	−ve	13 ± 0.4	9 ± 0.0	8 ± 0.0	11 ± 0.4	15 ± 0.1
**5b**	15 ± 1.0	9 ± 0.0	−ve	19 ± 0.0	19 ± 1.1	11 ± 0.6	−ve	14 ± 0.3	9 ± 0.0	8 ± 0.0	10 ± 0.3	15 ± 0.7
**5c**	15 ± 0.8	9 ± 0.0	11 ± 0.4	21 ± 0.7	13 ± 0.3	−ve	−ve	9 ± 0.0	9 ± 0.0	17 ± 0.4	8 ± 0.0	18 ± 0.3
**5d**	16 ± 0.2	18 ± 1.3	11 ± 0.0	14 ± 0.3	14 ± 0.8	11 ± 0.3	−ve	11 ± 0.3	9 ± 0.0	8 ± 0.0	14 ± 0.2	8 ± 0.0
**5e**	11 ± 0.8	11 ± 0.0	−ve	−ve	13 ± 0.44	−ve	−ve	9 ± 0.2	9 ± 0.0	15 ± 0.7	14 ± 1.1	9 ± 0.5
**5f**	9 ± 0.0	11 ± 0.6	13 ± 1.6	16 ± 0.5	14 ± 0.0	−ve	−ve	18 ± 0.5	15 ± 0.7	8 ± 0.0	18 ± 0.0	15 ± 1.6
**5g**	9 ± 0.4	9 ± 0.5	11 ± 0.0	14 ± 0.4	15 ± 1.0	−ve	−ve	9 ± 0.0	9 ± 0.0	17 ± 0.1	18 ± 0.0	14 ± 1.0
**5h**	22 ± 1.6	14 ± 0.3	11 ± 0.5	14 ± 0.4	14 ± 0.3	−ve	11 ± 0.7	11 ± 0.6	9 ± 0.0	11 ± 0.1	25 ± 1.6	16 ± 0.5
**5i**	13 ± 0.6	12 ± 0.9	−ve	19 ± 0.0	17 ± 0.6	−ve	−ve	14 ± 0.5	16 ± 0.2	8 ± 0.0	11 ± 0.2	18 ± 1.2
**5j**	12 ± 0.6	11 ± 0.2	−ve	9 ± 0.2	13 ± 0.2	−ve	11 ± 0.2	9 ± 0.7	9 ± 0.0	16 ± 0.5	13 ± 0.3	14 ± 0.5
**5k**	14 ± 0.4	11 ± 0.5	−ve	9 ± 0.0	14 ± 0.0	−ve	−ve	9 ± 0.6	9 ± 0.1	8 ± 0.0	13 ± 1.0	16 ± 0.4
**5l**	15 ± 0.4	9 ± 0.8	−ve	9 ± 0.0	14 ± 0.0	−ve	−ve	12 ± 0.8	9 ± 0.0	8 ± 0.0	15 ± 0.7	14 ± 0.5
**AMP**	30 ± 0.0	−ve	36 ± 0.7	−ve	45 ± 1.0	32 ± 0.4	18 ± 0.4	35 ± 1.0	30 ± 0.5	ND	ND	ND
**FLC**	ND	ND	ND	ND	ND	ND	ND	ND	ND	21 ± 0.5	16 ± 0.8	15 ± 0.0

**Table 2:** Minimum Inhibitory Concentrations (MICs) of the title compounds **5a–l**, AMP, and FLC against Gram-positive bacteria, Gram-negative bacteria, and fungi*.*

MIC Values (μg/mL)													
Compound No.	Strain Name												
	Gram-Positive Bacteria				Gram-Negative Bacteria						Fungi		
	*B.* *Subtilis*	*E.* *Fecalis*	MRSA	*S.* *Aureus*		*E.* *Coli*	*K. Pneumonia*	*P. Vulgaris*	*Ps. Aeruginosa*	*S. Enteridis*	*A.* *Niger*	*C. Albicans*	*P. Notatum*
**5a**	500	500	500	125		125	500	250	125	500	250	250	125
**5b**	125	500	500	62.5		125	500	250	250	250	62.5	250	250
**5c**	250	500	500	3.9		125	500	250	125	125	250	250	250
**5d**	250	250	>1000	31.25		125	500	250	62.5	250	15.6	62.5	62.5
**5e**	250	500	500	500		125	500	250	250	250	250	125	125
**5f**	250	500	500	250		125	500	250	250	500	250	125	250
**5g**	250	500	500	250		125	500	250	250	250	125	31.25	125
**5h**	62.5	125	500	250		250	500	250	250	500	31.25	7.8	62.5
**5i**	500	1000	500	62.5		62.5	500	250	125	125	125	250	250
**5j**	500	500	500	500		125	500	250	125	500	125	250	250
**5k**	500	500	500	500		62.5	500	1000	125	250	250	250	250
**5l**	250	1000	500	500		125	250	125	125	250	250	250	250
**AMP**	15.6	>1000	<7.8	>1000		<7.8	250	500	3.9	1000	ND	ND	ND
**FLC**	ND	ND	ND	ND		ND	ND	ND	ND	ND	15.6	31.25	250

**Table 3:** Binding energies and inhibition constants results of compounds **5c** and **5h** with their target proteins.

Protein ID	Binding Energy (∆*E*) [kcal/mol]	Estimated Inhibition Constant (*K*_i_) [μM]	Bounded Residues
4DH6	–7.88	1.67	THR232, GLY230 and PRO70
1EA1	–7.28	4.63	ARG326, HIS392, GLN72, VAL395, and ASN102

## Supplementary Materials

Supplementary materials are available online.
