# Supplementary material for: Synthesis, Spectroscopic Identification and Molecular Docking of Certain N-(2-{[2-(1H-Indol-2-ylcarbonyl) hydrazinyl](oxo)acetyl}phenyl)acetamides and N-[2-(2-{[2-(Acetylamino)phenyl](oxo)acetyl}hydrazinyl)-2-oxoethyl]-1H-indole-2-carboxamides: New Antimicrobial Agents
Source: Molecules. 2018 Apr 29;23(5):1043. doi: 10.3390/molecules23051043 (PMC6102541; doi:10.3390/molecules23051043)
Supplement: Supplementary file 1 [file molecules-23-01043-s001.pdf]

Synthesis, Spectroscopic Identification and Molecular Docking of Certain *N*-(2-{[2-(1*H*-Indol-2-ylcarbonyl)hydrazinyl](oxo)acetyl} phenyl)acetamides and *N*-[2-(2-{[2-(Acetylamino)phenyl](oxo)acetyl} hydrazinyl)-2-oxoethyl]-1*H*-indole-2-carboxamides: New Antimicrobial Agents

Maha S. Almutairi[1], Azza S. Zakaria[2], Reem I. Al-Wabli[1], I. Hubert Joe[3], Ali S. Abdelhameed[1] and Mohamed I. Attia[1,4]*

[1]Department of Pharmaceutical Chemistry, College of Pharmacy, King Saud University, P.O. Box 2457, Riyadh 11451, Saudi Arabia

[2]Department of Microbiology and Immunology, Faculty of Pharmacy, Alexandria University, Alexandria 21500, Egypt

[3]Centre for Molecular and Biophysics Research, Department of Physics, Mar Ivanios College, Thiruvananthapuram 695 015, Kerala, India

[4]Medicinal and Pharmaceutical Chemistry Department, Pharmaceutical and Drug Industries Research Division, National Research Centre (ID: 60014618), El Bohooth Street, Dokki, Giza 12622, Egypt

1. Antimicrobial Activity

1.1. Antimicrobial Agents

Stock solutions (1,000 μg/mL) of ampicillin (AMP) (Sigma-Aldrich Co. St. Louis, MO, USA) and fluconazole (FLC) (Shouguang-Fukang Pharmaceutical Ltd. Shandong, China). AMP was used as positive control for bacteria and FLC was used for fungi. AMP was dissolved in water while the test compounds as well as FLC were prepared in 100% dimethyl sulfoxide (DMSO) and were diluted with sterile distilled water. The antimicrobial discs (containing 25 μg FLC or 10 μg AMP) were purchased from ROSCO (Neo-Sensitabs, Taastrup, Denmark) and were stored at –80 °C until used.

1.2. Media

The bacteria were slanted on Nutrient agar (Difco Laboratories, Detroit, MI, USA), yeast was slanted on Sabouraud dextrose agar (SDA) (Difco Laboratories, Detroit, MI, USA), and the fungi were slanted on the Potato dextrose agar (PDA) medium (Eiken Chemical Co. Ltd. Tokyo, Japan). Muller Hinton broth (MHB) and Muller Hinton agar (MHA) were purchased from Sigma-Aldrich Co. (St. Louis, MO, USA) and were used following the manufacturer's instructions for the antimicrobial assay. Liquid RPMI 1640 medium supplemented with L-glutamine was purchased from Sigma-Aldrich Co. (St. Louis, MO, USA) and was added to 2% sodium bicarbonate and 0.165 M 3-(*N*-morpholino)propanesulfonic acid (MOPS) from Dojindo Laboratories (Kumamoto, Japan) then adjusted to pH 7.0 and was used for the assay of the yeast and moulds. MacConkey's agar, mannitol salt agar, cetrimide agar, and brain heart infusion broth (BHI) were obtained from Difco Laboratories (Detroit, MI, USA).

1.3. Culture Conditions

All clinical samples were first inoculated onto Sheep blood agar (SPML Co. Ltd, Riyadh, Saudi Arabia). The plates were incubated at 37 °C for 24-48 h. Identification of isolates was done according to the standard methods described elsewhere [1] and Clinical Laboratory Standards Institute [2]. Isolates were stored in BHI broth containing 16% (w/v) glycerol at −80 °C until further use.

1.4. Growth of the Tested Microorganisms

Staphylococcal isolates were re-inoculated onto mannitol salt agar and then the plates were incubated at 37 °C for 24-48 h. Mannitol fermentation was observed and recorded. Gram negative isolates were re-inoculated onto MacConkey's agar and then the plates were incubated at 37 °C for 24-48 h. Lactose fermentation was observed and recorded. *Ps. aeruginosa* strains were further re-inoculated on cetrimide agar at 37 °C for 24 h.

1.5. Disk Diffusion Assay

The antibacterial and antifungal screenings were conducted by the disk diffusion agar methods as described previously [3]. Bacterial and fungal suspensions were adjusted to a 0.5 McFarland standard corresponding to 5×10^6 CFU/mL. 100 μL

aliquot of each isolate suspension were uniformly spread onto MHA and SDA plates for bacteria and fungi, respectively. All test samples were dissolved in DMSO at 1000 µg/mL concentration, AMP (10 µg) was used as a positive control for bacteria and FLC (25 µg) was used as a positive control for fungi. Sterilized paper discs with only DMSO were used as negative controls for both bacteria and fungi. Plates were incubated under aerobic conditions at 35 °C for 24 and 48 h for bacteria and fungi, respectively. To obtain comparable results, all prepared solutions were treated under the same conditions. The experiments were carried out in triplicate. Plates were examined for evidence of antimicrobial activities, represented by a zone of inhibition of microorganism's growth around the discs, and diameters of clear zones were expressed in millimeter (mm) [4].

1.6. Determination of Minimum Inhibitory Concentrations (MICs)

The MIC of AMP and/or the synthesized compounds **5a-l** against the bacterial isolates were determined with a microdilution test, according to the reference method of the CLSI [5]. Stock solution of AMP pure drug was prepared in sterile distilled water while stock solution of each of the samples under test was prepared in DMSO to reach an initial concentration of 1000 µg/mL. Preparation of inocula for broth microdilution testing was performed in accordance with CLSI standard procedures [6] and the MIC was defined as the lowest concentration of the antibiotic or the test sample that prevented bacterial growth. Preparation of fungal inocula for the broth microdilution testing was performed in accordance with CLSI documents M27-A3 [7] and M38-A2 [8] with RPMI 1640 medium buffered to pH 7.0 with 0.165 M MOPS buffer for all organisms. In case of yeasts, the MICs were recorded as the lowest concentration at which a 50% decrease in turbidity relative to the turbidity of the growth control was observed, In case of the filamentous fungi, the MICs of the test samples and FLC were recorded as the lowest concentrations at which a prominent decrease in turbidity was observed.

References

1. Garcia, L.S. Clinical microbiology procedures handbook, American Society for Microbiology Press, Washington, DC, 2010.
2. Clinical and Laboratory Standards Institute (CLSI), *Abbreviated Identification of Bacteria and Yeas*, CLSI document M35-A2, Clinical and Laboratory Standards Institute (CLSI),Wayne, Pa, USA, 2nd edition, 2008.
3. National Committee Clinical Laboratory Standards, 2011. Performance standards for antimicrobial disk susceptibility tests. M100-S21, Vol. 31, No. 1.
4. Clinical and Laboratory Standards Institute (CLSI), *Performance Standards for Antimicrobial Disk Susceptibility Tests*, CLSI Document M02-A12, CLSI, Wayne, Pa, USA, 12th edition, 2015.

5. National Committee for Clinical Laboratory Standards, *Methods for Determining Bactericidal Activity of Antimicrobial Agents; Approved Guideline*, NCCLS document M26-A,Wayne, Pa, USA, 1999.
6. Clinical Laboratory Standards Institute (CLSI), "Methods for dilution antimicrobial susceptibility tests for bacteria that grow aerobically," CLSI Document M07-A10, CLSI, Wayne, Pa, USA, 2015.
7. Clinical and Laboratory Standards Institute (2008a) Reference method for broth dilution antifungal susceptibility testing of yeasts; approved standard-third edition; CLSI document M27-A3. Clinical and Laboratory Standards Institute, Wayne, Pa, USA.
8. Clinical and Laboratory Standards Institute (2008b) Reference method for broth dilution antifungal susceptibility testing of filamentous fungi; approved standard CLSI document M38-A2. Clinical and Laboratory Standards Institute, Wayne, Pa, USA.
